# Raman spectroscopy and advanced mathematical modelling in the discrimination of human thyroid cell lines

**DOI:** 10.1186/1758-3284-1-38

**Published:** 2009-10-28

**Authors:** Andrew T Harris, Manjree Garg, Xuebin B Yang, Sheila E Fisher, Jennifer Kirkham, D Alastair Smith, Dominic P Martin-Hirsch, Alec S High

**Affiliations:** 1 Department of Oral Biology, Level 6 Worsley Building, University of Leeds, Clarendon Way, Leeds, LS2 9LU, UK; 2 Avacta Group plc, York Biocentre, York Science Park, York, UK; 3 Section of Experimental Therapeutics, Leeds Institute of Molecular Medicine, University of Leeds, Leeds, UK; 4 School of Health Studies, University of Bradford, Bradford, UK; 5 Department of Ear Nose and Throat/Head and Neck Surgery, Calderdale and Huddersfield NHS Trust, Huddersfield, UK; 6 Department of Pathology Leeds Dental Institute, University of Leeds, Leeds, UK

## Abstract

Raman spectroscopy could offer non-invasive, rapid and an objective nature to cancer diagnostics. However, much work in this field has focused on resolving differences between cancerous and non-cancerous tissues, and lacks the reproducibility and interpretation to be put into clinical practice. Much work is needed on basic cellular differences between malignancy and normal. This would allow the establishment of a clinically relevant cellular based model to translate to tissue classification. Raman spectroscopy provides a very detailed biochemical analysis of the target material and to 'unlock' this potential requires sophisticated mathematical modelling such as neural networks as an adjunct to data interpretation. Commercially obtained cancerous and non-cancerous cells, cultured in the laboratory were used in Raman spectral measurements. Data trends were visualised through PCA and then subjected to neural network analysis based on self-organising maps; consisting of *m *maps, where *m *is the number of classes to be recognised. Each map approximates the statistical distribution of a given class. The neural network analysis provided a 95% accuracy for identification of the cancerous cell line and 92% accuracy for normal cell line. In this preliminay study we have demonstrated th ability to distinguish between "normal" and cancerous commercial cell lines. This encourages future work to establish the reasons underpinning these spectral differences and to move forward to more complex systems involving tissues. We have also shown that the use of sophisticated mathematical modelling allows a high degree of discrimination of 'raw' spectral data.

## Introduction

A range of optical methodologies including fluorescence, Fourier transform infrared and Raman spectroscopies have attracted much interest in biomedicine because of their potential advantages in offering non-invasive, rapid and objective diagnostics. Applications are being tested in such fields as microbial identification and cancer detection [1-10]. In cancer detection, research has focused on the potential to discriminate and resolve differences between cancer and normal tissues [11-13]. However, much of this work lacks the reproducibility and interpretation that would enable spectroscopy diagnostics to translate, 'from the bench to the bedside'. In order to translate this technique effectively to clinical practice much work is needed on basic cellular differences between cancerous and normal cells. Once these are appreciated, translating the work through to tissue would have a higher impact.

Raman spectroscopy has the highest specificity for chemical composition of target material amongst optical techniques. This, along with the relatively short spectral collection time, which can range from seconds to minutes, offers the possibility of rapid and sensitive diagnosis. Raman spectroscopy could therefore be potentially used to detect cancer at a biomolecular level prior to the morphological changes that the pathologist currently relies upon to make a diagnosis; making this technique extremely advantageous for early intervention. Raman spectroscopy relies on laser light (photons) interacting with molecules within the target material, causing them to vibrate. As a result, the photons are 'scattered' resulting in a frequency shift that is related to the energy of specific molecular vibrations. These vibrations are specific for particular molecular bonds and thus a biochemical 'fingerprint' of the target material can be established.

Biological cells are a complex mixture of molecules including proteins, nucleic acids, lipids and sugars enclosed within a membrane which is of itself a complex structure at the bio molecular level. The concentrations of these molecular constituents will vary within the cell; between cells of the same type with differing stages of growth and physiological function and between different cell types. This application of Raman spectroscopy as a diagnostic tool is therefore difficult, as its high biochemical specificity will detect all of these intra- and inter- cellular differences, giving complex backgrounds against which any diagnostic discrimination on the basis of disease-related changes must be made. Therefore, it is paramount that initial exploratory work to evaluate Raman spectroscopy as a diagnostic tool is undertaken on well characterised cells cultured under standardised laboratory conditions. Once spectral differences are understood using these simple systems, experimental work can shift to tissues where other factors such as blood and connective tissue will interfere with signals. Establishment of a clinically relevant cell-based model is therefore an important first step in this incremental process.

In order to obtain as much information as possible from the Raman spectra it is necessary to have an analysis tool capable of detecting small variations in spectra. Multivariate analysis methods such as Principal component analysis (PCA) have been employed[14] and indeed were used this study. However, PCA essentially rotates and scales the data allowing information to be lost in this scaling process. If differences in systems are large then this causes no problem. The possibility is that cellular biochemical differences between cancer and normal may be subtle; especially when dysplasia and very early changes are considered. PCA could potentially miss these subtle changes and therefore more advanced mathematical modelling systems are needed to interrogate the data. Neural networks are essentially non-linear statistical data modelling tools which find patterns in data[15]. The clear delineation between neural networks and computing are that functions are preformed collectively in a parallel series by the neurones, whereas basic computing relies on subtasks performed by individual units. By this rational neural networks are capable of learning, analogous with artificial intelligence. In order to optimise results from this technique, the system is 'trained' with data prior to test data being applied to the system. This system can appreciate small variations in datasets making it extremely advantageous in spectroscopic analysis.

Thyroid cancer is the most common endocrine malignancy[16]. The usually clinical presentation is with a neck mass, which may occasionally cause compression of the trachea, leading to respiratory embarrassment. The disease generally affects young females although an aggressive variant occurs in the elderly population and carries a very poor prognosis[17]. The diagnosis of thyroid cancer can be fraught with uncertainty. Initially a fine needle aspiration of the lump is undertaken by the clinician. This may not give adequate results due to sampling error or as in the case of follicular disease no comment can be made on tissue architecture or invasion; meaning further tissue is needed for certain accuracy. In cases where the lump is small or difficult to locate, the fine needle aspiration may have to be undertaken with ultrasound guidance. When cytological results prove inadequate; diagnosis is confirmed on excision biopsy when part of the gland is removed. Results from this biopsy usually take 2 to 3 weeks. Once cancer is diagnosed patients may have to undergo a second operation to remove the remainder of the thyroid. Spectroscopy would greatly speed up the diagnostic process whether pre-operatively or in the theatre setting; also a pre-operative definitive diagnosis would prevent the morbidity and possible mortality from a second operation.

The aim of this study was to identify whether Raman spectroscopy combined with advanced mathematical modelling (neural networks) could discriminate between 2 commercial thyroid cell lines; an anaplastic cancer variety and a 'normal' variety.

## Materials and methods

### Cell culture and preparation for spectroscopy

Human thyroid follicular epithelial cells (Nthy-ori 3-1), (a 'normal' commercial cell line) and human thyroid anaplastic carcinoma cell line (8305C) were obtained from the European Collection of Cell cultures (ECACC). The 'normal' cell line was originally obtained from normal adult thyroid tissue and transfected with a plasmid encoding for the SV40 large T gene[18]. These cells were cultured in RPMI (Sigma, USA), along with 5% L-glutamine (Sigma, USA), and 10% Foetal Calf serum (FCS). The anaplastic cells were originally established from an undifferentiated carcinoma in a female patient[19]. These cells were grown in Minimum Essential Medium Eagle (EMEM) with Hank's Salts (HBSS) (Sigma, USA), with 5% L-glutamine (Sigma, USA), 1% non - essential amino acids (Sigma, USA), and 10% FCS. Both cell lines were maintained in a 5% carbon dioxide incubator at 37°C. Prior to the acquisition of spectra, the cells were washed with PBS (phosphate buffered saline) 3 times, followed by suspension in 10% formalin for fixation for 10 minutes. Once fixed, the cells were re-suspended in PBS. A sample of PBS containing suspended cells was then pipetted onto a quartz slide and allowed to air dry. Once air dried Raman spectroscopy was performed.

### Raman spectroscopy

Raman spectra were obtained using a Renishaw 'System 1000' Raman microscope. Excitation was provided by a Sacher Lasertechnik Littrow external cavity laser set at 783 nm. Detection of the Raman scattered light was performed with a Renishaw RenCam NIR enhanced CCD camera. This camera is thermoelectrically cooled. The spectrometer was attached to a Leica DMLM microscope and the scattered light collected from the sample, via a 50× microscope objective. The spectrometer used holographic notch filters to remove Rayleigh scattered light from the collected light. The Raman scattered light was then dispersed across the CCD array detector by a single stage, 250 mm focal length grating spectrometer. The microscope was equipped with a motorised XYZ positioning stage (Prior) with integrated position sensors on the X and Y axes (Renishaw). Instrument control and data collection was performed with Renishaw WiRE software which operates within Galactic GRAMS software. Data acquisition time was 20 seconds for each cell.

### Data Analysis

Initially descriptive statistics were used to visualise the spectral graphs with Principal Component Analysis (PCA) to allow visualisation of the data trend. A neural architecture based on self-organising maps (SOM) was developed in VC++ to classify normal and anaplastic cells.

### Neural Architecture

A self-organising map (SOM) (20) is extremely useful as a nonparametric classifier due to its unsupervised residual plasticity. It classifies input patterns into groups based on the similarity between the patterns. Euclidean distance is used as a distance metric in SOM. A self-organising map is a single layer feed forward network where the output nodes are arranged in low dimensional grid. Each input is connected to all output neurons. Attached to every neuron there is a weight vector with the same dimensionality as the input vectors. The number of input dimensions is usually much higher than the output grid dimension.

We used SOM in a supervised manner and the neural architecture developed consists of *m *maps, where *m *is the number of classes to be recognised because of computational simplicity. Each map approximates the statistical distribution of a given class. This allows a self-adjusting process to be carried out by all the neurons in each local map and preserves the self-organisation paradigm by considering as many maps (*j *= 1,..., *m *where *m *is the number of classes) as the various classes which are taken into consideration to accomplish the classification task. In the training phase, each network is trained with observations belonging to an individual class in parallel. As we have two classes (Nthy-ori 3-1/8305C) the system consists of two maps and each map was 'trained' to recognise the Nthy-ori 3-1 and 8305C cells line respectively, using one-third of the original data set using VC++ software system.

**Figure 1:**
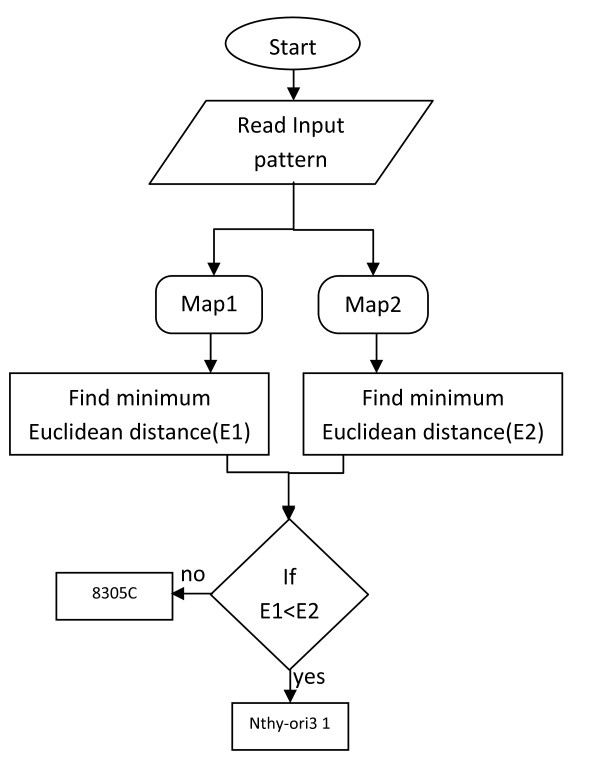
**Flow chart illustrating the testing phase of the neural network system for two classes where Map1 and Map2 are two SOMs trained on Nthy-ori 3-1 and 8305C cells respectively**.

Once the network is trained, a testing phase can take place where autonomous classification is carried out. At a certain time step *t*, a measurement vector *x *from the remaining data is presented to the network. The Euclidean distance measure is computed over all neurons of both the maps and the winner map (with the minimum distance) is considered as the estimated recognised class. Figure 1 illustrates the flow chart of the testing phase of the system containing two SOMs Map1 and Map2 trained for two classes Nthy-ori 3-1 and 8305C respectively.

## Results

**Figure 2:**
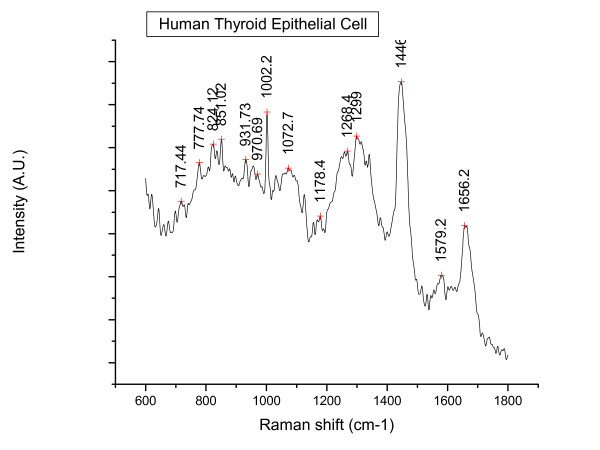
**A typical Raman spectrum from the Human thyroid epithelial cell (Nthy-ori 3-1); a non-cancerous cell line**.

**Figure 3:**
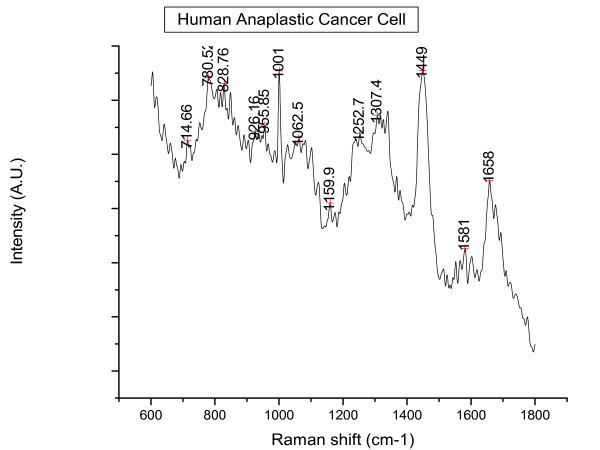
**A typical Raman spectrum from a Human anaplstic thyroid cancer cell (8305C); a cancerous cell line**.

**Figure 4:**
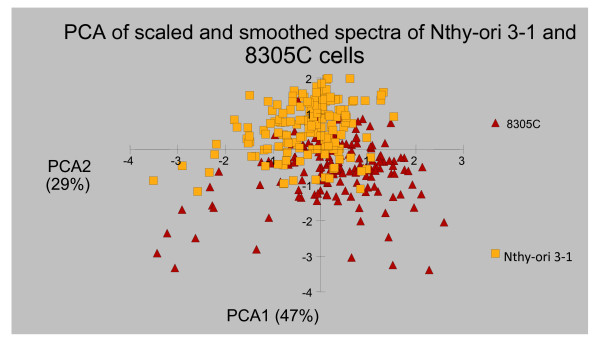
**The results of PCA comparing the non-cancerous (Nthy-ori 3-1) and cancerous (8305C) cell lines from the Raman spectra results**.

In total 52 spectra were obtained from the Nthy-ori 3-1 cells and 64 spectra from the 8305C cells. Figures 2 and 3 below illustrate a typical Raman spectral graph from the non-cancerous and cancerous thyroid cell lines. Figure 4 is the PCA plot of cancerous and non-cancerous Raman data. The PCA analysis where the 1^st ^principal component incorperated 47% of the variance and the 2^nd ^component 26%; (total of 76% variance for the first 2 components), is not totally discriminatory yet does show a distinct clustering of normal and cancerous cell lines but the overlap is too great to be diagnostic. Table 1 highlights the neural network result for the Raman data, providing a 95% sensitivity for the cancerous cell line and 92% sensitivity for normal cell line.

**Table 1:** Table illustrating the number of cells classified as non-cancerous or cancerous based on the neural network data.

	**Human Thyroid Epithelial Cells** **(Non-cancerous cell line)**	**Human anaplastic cell line** **(Cancerous cell line)**
**Human thyroid epithelial cells** **(Non-cancerous)**	48	4

**Human anaplastic cells** **(cancerous)**	3	61

The Out of 52 human thyroid epithelial cells 48 were correctly classified; 4 were incorrectly classified as being cancerous. When the 64 anaplastic cancerous cells similarly analysed, 3 were wrongly classified as normal. algorithm was correct for 95% of the cancerous cell line and 92% for the normal cell line.

## Discussion

Our study has demonstrated that Raman spectroscopy, coupled with neural network analysis is able to discriminate between cancer and non-cancer cells in a simple model system with a high degree of accuracy. The results of the neural network demonstrate a clear distinction between the 2 cohorts 95%, and 92% sensitivity. The obvious peak differences at 780 nm and 830 nm from previous literature are thought to correspond to DNA: O-P-O backbone stretching and nucleic acids [20-22]. It would be expected that cancerous cells would have a greater amount of nuclear matter due to the increased mitosis they undergo. The greater peak intensity in the 1656/8 region in the cancer cohort is attributed to the Amide I: α-helix.

Similar work has been reported by Crow and colleagues in 2005[23]. They used Raman spectroscopy and a diagnostic algorithm to differentiate prostatic carcinoma cell lines. In their study, the cells were cytospun and the pellet placed on a calcium fluoride slide for spectroscopic analysis. Therefore; the spectra were collected from a pellet rather than single cells. Their results proved highly accurate with sensitivities of 98% and their findings correlate with ours in that nucleic acid components, DNA backbone and α-helix proteins differ between the malignant groups. Their work did not have a non-cancerous cell line so direct comparisons with this study are impossible, yet differing degrees of malignant aggressiveness were correlated with changes in basic biochemical properties in the regions demonstrated in this study.

Jess and co-workers (2007) studied Raman spectroscopy in differentiating cervical cells[21]. Spectra were compared from a normal human keratinocyte cell line and a cancerous line. The primary human keratinocyte line was then infected with a virus containing the gene for HPV 16 E7, and further spectra taken to discriminate between similar cells expressing differing proteins. PCA was used for discriminatory purposes and this gave >90 sensitivity for live cells and slightly higher for a 'fixed' cohort.

In this study PCA illustrated a definite localisation of each cohort but this would not be significant enough for diagnostic purposes. However, neural network analysis provided a superior analytical tool with its greater than 90% accuracy for either cell line. Whilst cancer versus non-cancer is being analysed multivariate statistical methods such as PCA, linear discriminant analysis and classical least square fitting have been shown to confer high accuracy[14]. However, Raman spectroscopy is highly specific in detailing biochemical composition, it is therefore necessary to have similar precision in 'un-locking' the data, otherwise subtle changes such as those seen in the progression of dysplastic tissue to carcinoma may well be missed. In this study neural network analysis conferred greater accuracy than PCA in cell line discrimination; and this may well need to be the tool of choice when complex systems such as tissues are analysed.

## Conclusion

In this preliminay study we have demonstrated an ability to successfully distinguish between "normal" and cancerous commercial cell lines. This encourages future work to establish the reasons underpinning these spectral differences and to move forward to more complex systems involving tissues. We have also shown that the use of sophisticated mathematical modelling allows a high degree of discrimination of Raman data.

## Competing interests

The authors report no conflicts of interest. The authors alone are responsible for the content and writing of the paper.

## Authors' contributions

ATH was invloved with cell culture and Raman data collection. MG undertook the PCA and neural network analysis of the Raman data. JK, XB provided support for cell culture. DAS provided support for the Raman system. JK, XB, DAS, SEF, ASH provided support for the methodology. DPMH, SEF and ASH provided input for the clinical application to thyroid surgery. All authors had an editorial contribution. All authors read and approved the final manuscript.
